# A low-dose immunotherapy targeting Fc gamma Receptors and heparan sulfate proteoglycan to impact myeloid cells and control cancers with diverse immunosuppressive profiles

**DOI:** 10.3389/fimmu.2026.1734778

**Published:** 2026-07-23

**Authors:** Nora Kakwata-Nkor Deluce, Elodie Creton, Alexandra Savatier, Honorine Lucchi, Laureen Bardouillet, Oscar Pereira Ramos, Philippe Berthon, Michel Léonetti

**Affiliations:** 1 Blue Bees Therapeutics, Parc Club-Orsay Université, Orsay, France; 2 Département Médicaments et Technologies pour la Santé, Université Paris Saclay, CEA, INRAE, SPI, Gif-sur-Yvette, France

**Keywords:** dendritic cell activation, Fc-gamma receptor, FcγR/HSPG-engaging immunotherapy, heparan-sulfate proteoglycan, highly immunosuppressed tumors, myeloid-targeted immunotherapy

## Abstract

Myeloid cells play a key role in cancer-associated immunosuppression because their accumulation and reprogramming inhibit antitumor responses and support tumor growth. To modulate their activity, we targeted Fcγ receptors (FcγRs), which are broadly expressed in myeloid subsets. Since low-affinity Fcγ receptor IIb (FcγRIIb) mediates inhibitory signaling, we designed an immunotherapy active at a low dose to limit binding to FcγRIIb while retaining interaction with higher-affinity FcγRs. We engineered an Fc-based fusion protein, whose activity is potentiated by its ability to engage both FcγRs and a coreceptor, Heparan Sulfate Proteoglycan (HSPGs). This immunotherapy, named Fc-T54, combines an HSPG-ligand, named T54, with human IgG1-Fc. Compared with Fc, Fc-T54 displayed superior binding to Fcγ receptor IIa (FcγRIIa), Fcγ receptor IIIa (FcγRIIIa) and enhanced interactions with human leukocytes, including neutrophils, B-lymphocytes, as well as with monocytes, and dendritic cells (DCs) within peripheral blood mononuclear cells. Functionally, Fc-T54 increased monocyte/macrophage and B-cell numbers, reduced neutrophil abundance, and boosted DC activation in both the human and murine systems. Subcutaneous administration of low-dose Fc-T54 - or its murine surrogate - inhibits tumor growth in immune-deserted and immune-excluded mouse models and synergizes with anti-PD-1 therapy in an immune-inflamed model. Tumor microenvironment analysis in the bladder cancer model revealed that the immunotherapy decreased the proportion of granulocytic myeloid-derived suppressor cells while increasing CD8+ T cells and natural killer cells, promoting a microenvironment more prone to tumor control. This FcγR/HSPG-engaging immunotherapy, administered subcutaneously, offers a novel approach to modulate the myeloid compartment and improve outcomes for ICI-resistant, deserted/excluded tumors, and for inflamed tumors when used in combination regimens.

## Highlights

An Fc fusion protein is engineered to engage both FcγRs and HSPGs for myeloid cell modulation. Its low-dose subcutaneous injection in mice reduces tumor growth in cancers with distinct immunosuppressive profiles and remodels the tumor microenvironment.

## Introduction

Myeloid cells are key mediators of cancer immunosuppression, shaping a tumor-permissive microenvironment by inhibiting T cells, secreting cytokines, and recruiting regulatory networks (1, 2). Immunosuppressive myeloid populations include myeloid-derived suppressor cells (MDSCs) (3), tumor-associated macrophages (TAMs) polarized toward an immunosuppressive phenotype (4), and dysfunctional dendritic cells (DCs) that fail to prime antitumor immunity and expand regulatory T cells (5). Current therapies targeting these cells, such as CSF-1R inhibitors or CD40 agonists, can enhance the efficacy of checkpoint blockade by reprogramming TAMs or by reducing MDSC infiltration. However, these approaches face limitations including incomplete myeloid depletion, enrichment of resistant myeloid subsets, compensatory neutrophil populations, and adverse events (6–9). Therefore, novel therapeutic strategies that overcome these resistance mechanisms and elicit durable responses are needed, particularly in highly immunosuppressed cancers characterized by abundant MDSCs and resistance to current immunotherapies (7, 10, 11). This prompted us to develop a new immunotherapy that targets myeloid cells.

All myeloid cell populations that arise from progenitor cells express, in a variable manner, Fc gamma receptors (FcγRs) that can modulate their activity through mechanisms initiated upon binding of the Fc domain of IgGs (12). Although FcγRs are targeted by numerous mono-, bi-, and tri-specific antibodies developed to date (13), they have never been considered as a sole immunotherapeutic target. Essentially, because this family (14) has activating receptors, such as Fcγ receptor I (FcγRI), FcγRIIa and FcγRIIIa, but also inhibitory receptors, mostly FcγRIIb, that could thwart the immunotherapeutic effect (15, 16). However, and interestingly, FcγRIIb is the member of the family with the lowest affinity for antibodies, with a KD of approximately 1–3 × 10^−5^ M. As a result, a non-specific antibody or Fc fragment used at concentrations below the KD for FcγRIIb would predominantly interact with activating FcγRs in myeloid cells. Nevertheless, such proteins, when in their monomeric forms, are generally inefficient at triggering cell signaling and, consequently, at modulating cellular activity (17, 18). Therefore, they must be modified to make them appropriate myeloid cell modulators.

Many soluble endogenous proteins, including cytokines, chemokines, and growth factors, modulate cell activity and numerous biological processes through the engagement of receptor and coreceptor (19). This mechanism of dual interaction is also shared by proteins of infectious microorganisms that use it to disseminate and/or alter immune defense mechanisms (20). Heparan sulfate proteoglycans (HSPGs) are glycoproteins ubiquitously distributed on the cell surface that often play the role of coreceptor (21). HSPG-binding proteins interact with the heparan sulfate (HS) groups of HSPGs via regions rich in basic residues (22). Previously, we showed that coupling a basic-rich HSPG-ligand to a protein antigen (Ag) enables an approximately 100- to 1000-fold decrease in immune complex concentrations required for presentation by antigen-presenting cells (APCs) via FcγRs to specific T-cells (23). In addition, we found that the fusion of such an HSPG-ligand to protein vectors targeting various receptors allows for an increase in the immune response *in vitro* (24, 25) and *in vivo* (26). These findings, which highlight the value of proteins capable of binding both a receptor and an HSPG coreceptor in specific immune responses, led us to investigate whether a fusion protein combining an IgG1-Fc fragment and an HSPG ligand, termed Fc-T54, could (i) efficiently target FcγR-expressing immune cells, (ii) modulate their activity, and (iii) impact tumor growth. Notably, ICI therapies currently used in patients are predominantly effective in tumors with inflamed characteristics and much less effective in those displaying deserted or excluded immune phenotypes (27, 28). Therefore, we evaluated whether Fc-T54 can affect tumor growth using a panel of four syngeneic mouse tumor models that vary in their sensitivity to ICIs and encompass a spectrum from deserted to inflamed phenotypes.

In this study, we describe the production of Fc-T54. We then characterized its binding properties to FcγRs and heparan sulfates as well as its interactions with both human and murine immune cells. Furthermore, we demonstrated the capacity of Fc-T54 to modulate immune cell activity and showed that when administered at low doses via the subcutaneous route, this fusion protein or its murine surrogate can inhibit tumor growth in syngeneic models with excluded or deserted immune phenotypes and, when used in combination regimens, in inflamed tumors. Additionally, we showed that Fc-T54 alters the TME in a manner that favors tumor control. Collectively, these findings indicate that Fc-T54 represents a novel strategy to modulate the myeloid compartment and broaden therapeutic options for ICI-resistant deserted/excluded tumors.

## Materials and methods

### Protein production

Fc-T54 and Fc were produced following the protocol for expression in Expi-HEK cells (Gibco Transfection Kit ExpiFectamine™293). Briefly, Expi-HEK cells (3 × 10^6^ cells/ml) were transfected with a pcDNA™3.4 plasmid encoding either Fc-T54 or Fc and ExpiFectamine™293 Reagent. After 16 h of incubation at 37 °C, medium was added. After four additional days of incubation, the supernatants were collected, filtered, and supplemented with a protease inhibitor cocktail. After dilution in 0.05% Tween-PBS, the proteins were immunopurified using a Protein A column (ProSep^®^-vA High-Capacity, Millipore). The eluate was neutralized with Tris-HCl (1 M, pH8), and subjected to gel filtration (AKTA HiLoad^®^ 16/600 Superdex^®^ 200pg, Cytiva). Proteins were analyzed by 4-12% SDS-PAGE gels. Proteins were stored at -20 °C. An identical protocol was used to generate a mouse IgG2a-Fc fragment, named mFc, and a mouse Fc-T54 surrogate, designated mFc-T54, in which Fc encoded murine IgG2a.

### Binding to heparin

To assess Fc-T54 and Fc binding to heparin, microtiter plates were coated with heparin-albumin (Hep-Alb) (1 µg/ml in 0.1 M phosphate buffer pH7.2) and saturated with 0.3% BSA (200 µL/well). Then, plates were washed and serial dilutions of Fc-T54 and Fc (0.1 M phosphate buffer, pH7.4, containing 0.1% BSA) were added to the wells. After 1-hour RT, plates were washed and 100 µL of Goat Anti-Human Horseradish Peroxidase conjugate (GAH-PO) was added. After 30 min of incubation, the plates were washed, and 200 µL of 2,2′-azino-bis 3-ethylbenzothiazoline-6-sulfonic acid (ABTS) was added. Absorbance was measured at 415 nm.

### Binding to FcγRI, FcγRIIIa, FcγRIIa and FcγRIIb/c receptors

Microtiter plates were coated with avidin (1 µg/ml in 0.1 M phosphate buffer pH7.2) and saturated with 0.3% BSA (200 µL/well) or coated with 0.3% BSA (300 µL/well).

To assess binding to FcγRI, both sets of plates were washed and incubated with serial dilutions of biotinylated FcγRI for 1 hour RT. After washings, fixed amounts of Fc-T54 or Fc (100 nM) were added. After one hour RT the plates were washed, and GAH-PO was added. After 30 min, the plates were washed and ABTS was added. Absorbance was measured at 415 nm. To eliminate non-specific binding, the optical signal from BSA-coated plates was removed from that of the avidin-coated plates.

To assess binding to FcγRIIIa, FcγRIIa, and FcγRIIb/c, serial dilutions of Fc-T54 or Fc proteins were pre-incubated with biotinylated FcγRIIIa, FcγRIIa (30 nM), or FcγRIIb/c (100 nM) overnight at 4 °C. The mixture was then transferred to avidin-coated plates. After one hour, the plates were washed, GAH-PO was added, and binding was assessed as for FcγRI.

To assess binding to heparin and FcγRI, serial dilutions of Fc-T54 or Fc were incubated in heparin-albumin- and BSA-coated wells. After 1-hour RT, plates were washed, and biotinylated-FcγRI (20 nM) was added. One hour later, plates were washed, streptavidin-PO was added. Thirty minutes later, plates were washed, and 200µl ABTS was added. Absorbance was measured at 415 nm.

To assess binding to heparin and FcγRIIIa, FcγRIIa, and FcγRIIb/c, a fixed amount of biotinylated FcγRIIIa, FcγRIIa, or FcγRIIb/c (100nM) was incubated with two different dilutions of Fc-T54 or Fc (100 and 300 nM for binding to FcγRIIIa; 300 and 1000 nM for binding to FcγRIIa and FcγRIIb/c, respectively) overnight at 4 °C. The mixtures were then transferred to heparin-albumin- and BSA-coated wells. After 1-hour RT, plates were washed and treated with GAH-PO and ABTS as for heparin/FcγRI assay.

### Binding to human leukocytes

To assess Fc-T54 and Fc binding to human leukocytes, fresh whole blood was collected from healthy donors (n= 6) and subjected to red blood cell lysis using RBC Lysis Buffer (Thermo Fisher Scientific, 00-4300-54). Leukocytes were plated at 5×10^5^ cells/well in V-bottom 96-well plates. Biotinylated Fc-T54 or Fc (10 or 30 nM) was added for 30 min at 4 °C. Cells were washed twice with PBS/0,5% BSA and subsequently stained with streptavidin-PE (Miltenyi; 1:300 dilution) together with a cocktail of fluorochrome-conjugated surface antibodies for myeloid or lymphoid immunophenotyping (see **Supplementary Table 1** for full antibody list). Samples were incubated 30 min at 4 °C in the dark, washed, and acquired on an Attune NxT Flow Cytometer (Thermo Fisher Scientific) and analyzed using FlowJo 10.9 software. Dead cells (Thermo Fischer Scientific, #L34966) and doublets (FSC-H vs FSC-A) were excluded prior to analysis. Leukocyte subpopulations were identified via sequential gating as follows: neutrophils (CD3^−^ CD66^+^ CD15^+^), monocytes (CD3^−^ CD11b^+^ HLA-DR^+^ CD14^+^) dendritic cells (CD14^−^ CD11b^+^ HLA-DR^+^ CD11c^+^), T cells (CD3+ CD19-), B cells (CD3^−^ CD19^+^) and NK cells (CD3^−^ CD56^+^). Binding was quantified as the percentage of PE-positive cells within each leukocyte subset.

### Binding to DCs

To assess Fc-T54 and Fc binding to DCs, the murine JAWSII DC line (ATCCCRL-11904) was used. Cells were seeded in 96-well plates (1×10^5^ cells/well) and incubated for 30 min at 4 °C with serial dilutions of Fc-T54, Fc, T-54, or a combination of Fc and T54. Following incubation, cells were washed and stained with streptavidin-PE (Miltenyi; 1:300 dilution). After 30 min at 4 °C, cells were washed and acquired on an Attune NxT Flow Cytometer (Thermo Fisher Scientific) and analyzed using FlowJo 10.9 software. Dead cells (Thermo Fischer Scientific, L34966) and doublets (excluded based on FSC-H Vs FSC-A) were gated out prior to analysis. Binding was quantified as the percentage of PE-positive cells within live cells. To evaluate binding specificity, Fc-T54 (100nM) was incubated with or without rat anti-mouse CD16/CD32 Ab (clone 2.4G2, 4 µg/mL), soluble heparan sulfate (HS) 3µM, or a combination of both reagents CD16/CD32 Ab and HS. HS was kindly provided by Dr Hugues Lortat-Jacob. The mixture was processed as described above, including washing, staining with GOAH−PE, flow cytometry acquisition, and data analysis.

### Activation of DCs and involvement of the Syk-tyrosine kinase pathway.

To assess the Activation of DCs and involvement of the SYK tyrosine kinase pathway, cells were seeded in 96-well plates (1×10^5^ cells/well) and stimulated for 24 h at 37 °C with Fc-T54, Fc, T54, or Fc+T54 (300 nM each), cells were then washed, stained with BV785-conjugated anti-mouse CD69 Ab (Biolegend 104543, dilution 1/50) and acquired on an Attune NxT Flow Cytometer (Thermo Fisher Scientific) and analyzed using FlowJo 10.9 software) Dead cells (Thermo Fischer Scientific, L34966) and doublets (FSC-H Vs FSC-A) were excluded prior to analysis. To assess the involvement of the syk-tyrosine kinase pathway, JAWSII cells were seeded in plates (1×10^5^ cells/well) and incubated for 30 min at 37 °C, with or without The SYK R406 (300 nM, Sigma). Next, Fc-T54 or Fc (300 nM) was added. After 24 h at 37 °C, Cells were processed as described above (washing, anti-CD69 staining, flow cytometry acquisition, and analysis). Activation was quantified as the percentage of CD69^+^ cells among live cells.

### Assessment of Fc-T54 activity *in vitro* using human leukocytes

To assess the ability of Fc-T54 to affect human immune cell activation, leukocytes were isolated from fresh whole blood of healthy donors (n=6) after red blood cell lysis using RBC Lysis Buffer Buffer (Thermo Fisher Scientific). Cells were seeded in plates (5x10^5^) cells/well in 200 µL, RPMI medium supplemented with 5% human albumin serum) and stimulated with Fc-T54 or Fc (300 nM or left unstimulated for 72 h (37 °C, 5% CO_2_). Cells were then washed twice with PBS + 0,5% BSA and stained, 30 min at 4 °C in the dark, with fluorochrome-conjugated antibodies for myeloid, lymphoid immunophenotyping markers plus activation markers CD69 and CD86 (see **Supplementary Table 1** for antibody panel). Samples were then washed, fixed (BD Cell fix #554655) and acquired on an Attune NxT Flow Cytometer (Thermo Fisher Scientific). Data were analyzed with FlowJo 10.9 software. Dead cells (Thermo Fischer Scientific, L34966) and doublets (FSC-H/A) were excluded prior to sequential analysis. Leukocyte subpopulations were identified via sequential gating as follows: neutrophils (CD3^−^ CD66^+^ CD15^+^), monocytes/macrophages (CD3^−^ CD11b^+^ HLA-DR^+^ CD14^+^, CD86+), dendritic cells (CD14^−^ CD11b^+^ HLA-DR^+^ CD11c^+^), T cells (CD3+ CD19-) were subdivided into CD8^+^ T cells, CD4^+^ T cells, B cells (CD3^−^ CD19^+^) and NK cells (CD3^−^ CD56^+^). The proportion of each leukocyte subpopulation was determined alongside their activation status. Activation was quantified as the frequency of CD69^+^ cells for all subsets and CD86^+^ cells for dendritic cells.

### Antitumor efficacy

Female C57BL/6 mice aged 6–8 weeks (Janvier Lab, France) were housed under controlled temperature and a 12-hour light/dark cycle, with food/water provided ad libitum in the animal facility of CEA. All procedures were performed according to the approved protocols (APAFIS#33447-2021101317231911v1). These were unblinded experiments: experimenters conducting procedures, assessing outcomes, and analyzing data were aware of group allocation. MB49 (Bladder Carcinoma; CVL_7076), B16F10 (melanoma; CRL_6475), MC38 (colon carcinoma; CVCL_B288), and PAN02 (pancreatic Adenocarcinoma; CRL_2553) cells. MB49 (0.5 or 1 M cells), MC38 (1 M cells), Pan02 (3 M cells), and B16F10 (0.2 M cells) cell lines were injected subcutaneously into mice. Three to seven days later, mice were randomized and injected s.c. with either mFc-T54, mFc, or Fc-T54 (0.02 nmol per mouse) or PBS (Control) once for MB49 and B16F10 models, or four times for Pan02 and MC38. The Anti-PD-L1 biosimilar Ab Avelumab (Ave) and the mouse anti-PD-1 Ab (clone RMP1-14) were injected intraperitoneally (1 nmol per mouse). Tumor volume was monitored using a caliper. Mice were monitored daily for signs of distress or illness. At the experimental endpoint animals were humanely euthanized in accordance with institutional guidelines. If animals reached a body score limit before the end of the experiment, they were euthanized and excluded from the tumor-size follow-up.

### Analysis of tumor-infiltrating immune cells

Immune cell analysis was performed on tumors of euthanized MB49 mice previously treated with PBS or Fc-T54. Tumors were harvested, mechanically disrupted, and digested with collagenase IV (2 mg/ml, Roche). Collagenase activity was stopped by adding 5 ml RPMI medium + 10% FBS. The cells were filtered using a 40 μm cell-strainer. Cells were then stained for 30 min at 4 °C with fluorochrome-conjugated antibodies specific for tumor-infiltrating immune cells (see **Supplementary Table 2** for antibody panel). Dead cells (Thermo Fischer Scientific, L34966) and doublets (FSC-H vs FSC-A) were excluded prior to analysis. Samples were acquired on an Attune NxT Flow Cytometer (Thermo Fisher Scientific) and analyzed using FlowJo v10.9 software. Populations were identified using hierarchical gating as detailed: granulocytic MDSC (G-MDSC: CD11b^+^Ly6G^+^Ly6C^low^); dendritic cells as CD11c^+^IAIE^+^; NK cells as CD3^−^NK1.1^+^; and T cells as CD3^+^. The proportion of each tumor-infiltrating immune cell subpopulation was determined.

## Results

### Production of a fusion protein with FcγR- and HS-binding ability

**Figure 1:**
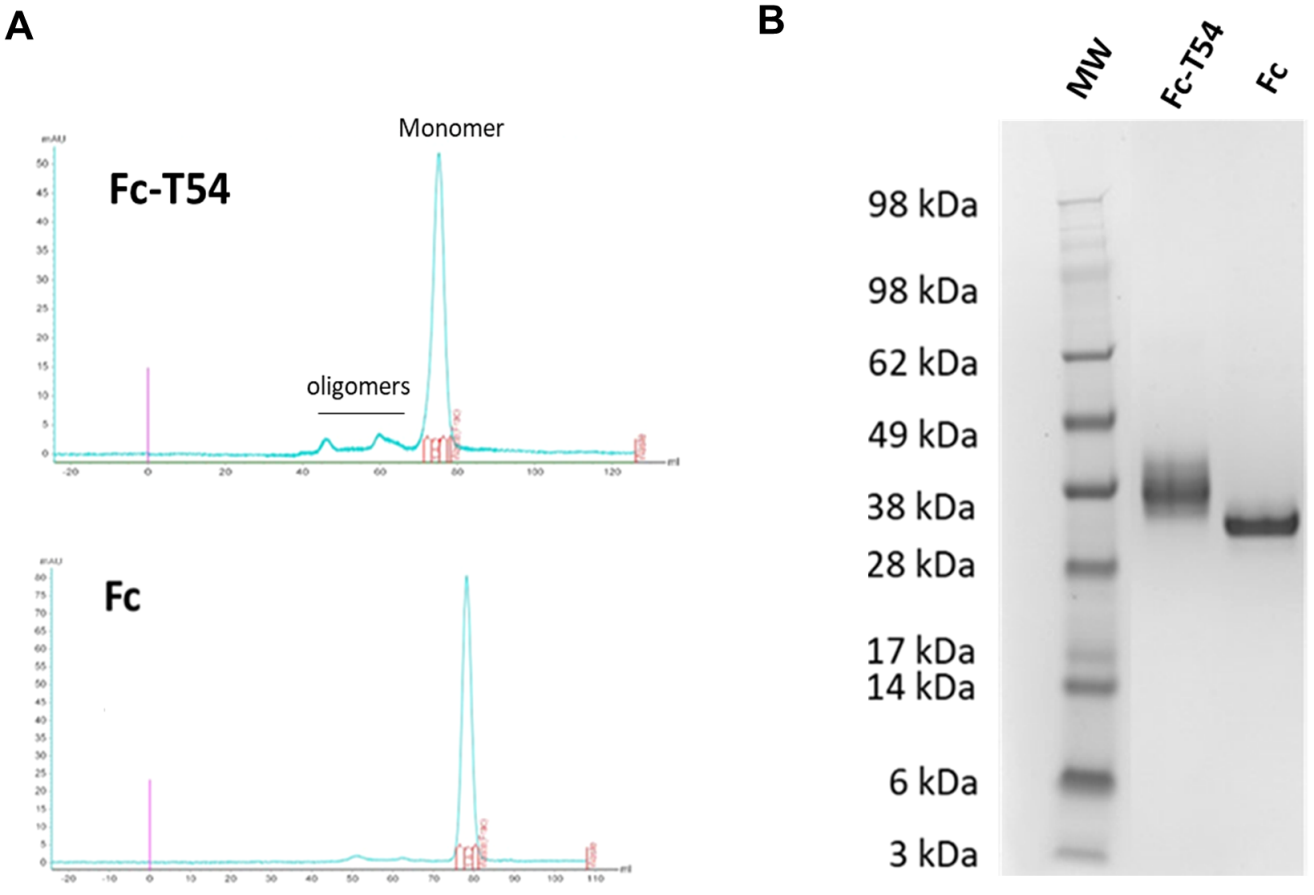
Characterization of purified Fc-T54 and Fc: integrity and homogeneity. **(A)** Size-exclusion chromatography (SEC) profile of Fc-T54 and of Fc. Immuno-purified Fc-T54 and Fc were purified by SEC to remove oligomers. **(B)** SDS-PAGE gel under denaturing conditions, showing markers of molecular weight (MW) and Fc-T54 and Fc after electrophoretic migration.

To assess whether targeting both FcγRs and a coreceptor can modulate the activity of immune cells expressing these receptors, we produced two recombinant proteins. The first, called Fc-T54, contains an Fc (Fc), a six-residues linker, and a Tat domain (T54) derived from the HIV transcriptional transactivator, which has a heparan sulfate (HS) binding site (29). The Tat domain differed from that described by Knittel and Coll (26). by the lack of three C-terminal basic residues. The second protein, called Fc, contains Fc and the linker domain. Fc-T54 and Fc were expressed in HEK cells, immunopurified on a protein A gel column, and subjected to size-exclusion chromatography to remove high molecular weight oligomers (**Figure 1A**). Gel electrophoresis showed that Fc-T54 migrated in a predominant band higher than that corresponding to Fc, indicating that T54 was fused to Fc (**Figure 1B**). For Fc-T54 and Fc the molecular weights found (38 kDa and 32 kDa respectively) are higher than their theoretical masses (30321.5 Da and 26078.5 Da) indicating post-translational modifications during expression in HEK cells.

**Figure 2:**
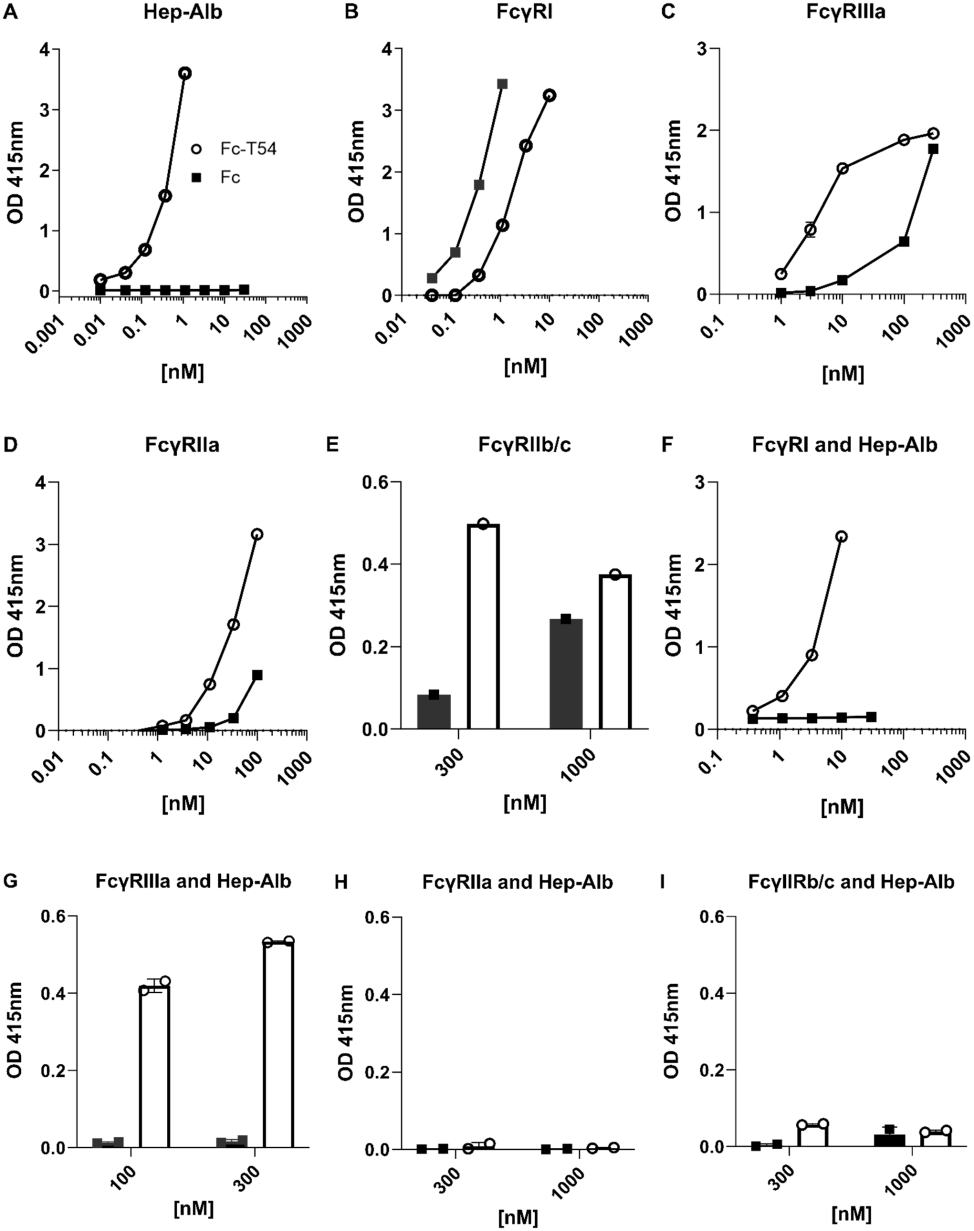
Binding of Fc-T54 and Fc to heparin and Fcγ receptors. **(A)** Binding of Fc-T54 and Fc proteins to heparin: Serial dilutions of Fc-T54 and Fc were incubated on heparin-albumin-coated plates. **(B)** Binding to FcγRI: Serial dilutions of Fc-T54 and Fc were incubated on avidin-coated plates. Then, biotinylated- FcγRI was added. **(C–E)** Binding to FcγRIIIa, FcγRIIa and FcγRIIb/c: Fc-T54 and Fc were pre-incubated overnight at 4 °C with biotinylated-FcγRIIIa, -FcγRIIa and -FcγRIIb/c, respectively. Then, the mixtures were added to avidin-coated plates. Binding to the plates in **(A–E)** was detected using peroxidase-coupled goat anti-human IgG (PO-GOAHIgG) and ABTS as substrate. **(F–I)** Study of simultaneous binding to Heparin and FcγRI, FcγRIIIa, FcγRIIa, FcγRIIb/c: **(F)** Serial dilutions of Fc-T54 and Fc were incubated on heparin-albumin-coated plates, followed by an incubation with biotinylated FcγRI. **(G–I)** Fc-T54 and Fc were pre-incubated with biotinylated-FcγRIIIa, -FcγRIIa, -FcγRIIb/c, respectively. Then, the mixtures were transferred to heparin-albumin-coated plates. Binding in **(F–I)** was detected using peroxidase-coupled streptavidin and ABTS as substrate.

To determine whether the T54 domain endows Fc with the ability to bind HS, Fc-T54 and Fc were compared using ELISA. Heparin, a sulfated polysaccharide representative of the HS family, was used. No optical signal was detected for Fc, indicating that it did not bind heparin (**Figure 2A**). In contrast, Fc-T54 produced a dose-dependent optical signal, demonstrating its capacity to bind to heparin.

### The HS-binding ability makes the Fc domain capable of interacting more efficiently with low-affinity human FcγRs and increases its ability to bind FcγR-expressing immune cells

To evaluate whether T54 conjugation affects the ability of Fc to bind human Fcγ receptors, Fc-T54 and Fc were assessed for their binding to soluble FcγRI, FcγRIIa, FcγRIIb, and FcγRIIIa. When mixtures of biotinylated FcγRI and serial dilutions of Fc-T54 or Fc were incubated on avidin-coated plates, dose-dependent optical signals were observed (**Figure 2B**). Notably, a lower concentration of Fc was required to achieve the same signal compared with Fc-T54, suggesting that the covalent coupling of T54 to Fc reduces affinity for FcγRI. Both Fc-T54 and Fc also exhibited dose-dependent binding to FcγRIIIa. However, in this case, Fc-T54 required a lower concentration than Fc to reach the same signal, suggesting that T54 conjugation enhances binding to FcγRIIIa (**Figure 2C**). A similar trend was observed for FcγRIIa and FcγRIIb, with increased binding observed for Fc-T54 compared with Fc. However, binding to these receptors was only detectable at higher concentrations for both Fc-T54 and Fc, which is consistent with the lower intrinsic affinity of the Fc domain for FcγRIIa and FcγRIIb (12).

Finally, we investigated the ability of Fc-T54 and Fc to bind to both heparin and FcγRI, FcγRIIa, FcγRIIb, or FcγRIIIa. No optical signal was detected for Fc (**Figures 2F–I**), in agreement with its inability to bind heparin (**Figure 2A**). A signal was measured for Fc-T54 with FcγRI or FcγRIIIa, demonstrating its ability to bind to these two receptors and heparin (**Figures 2F, G**). For FcγRIIa and FcγRIIb, the optical signal was too weak to determine whether simultaneous binding might occur.

**Figure 3:**
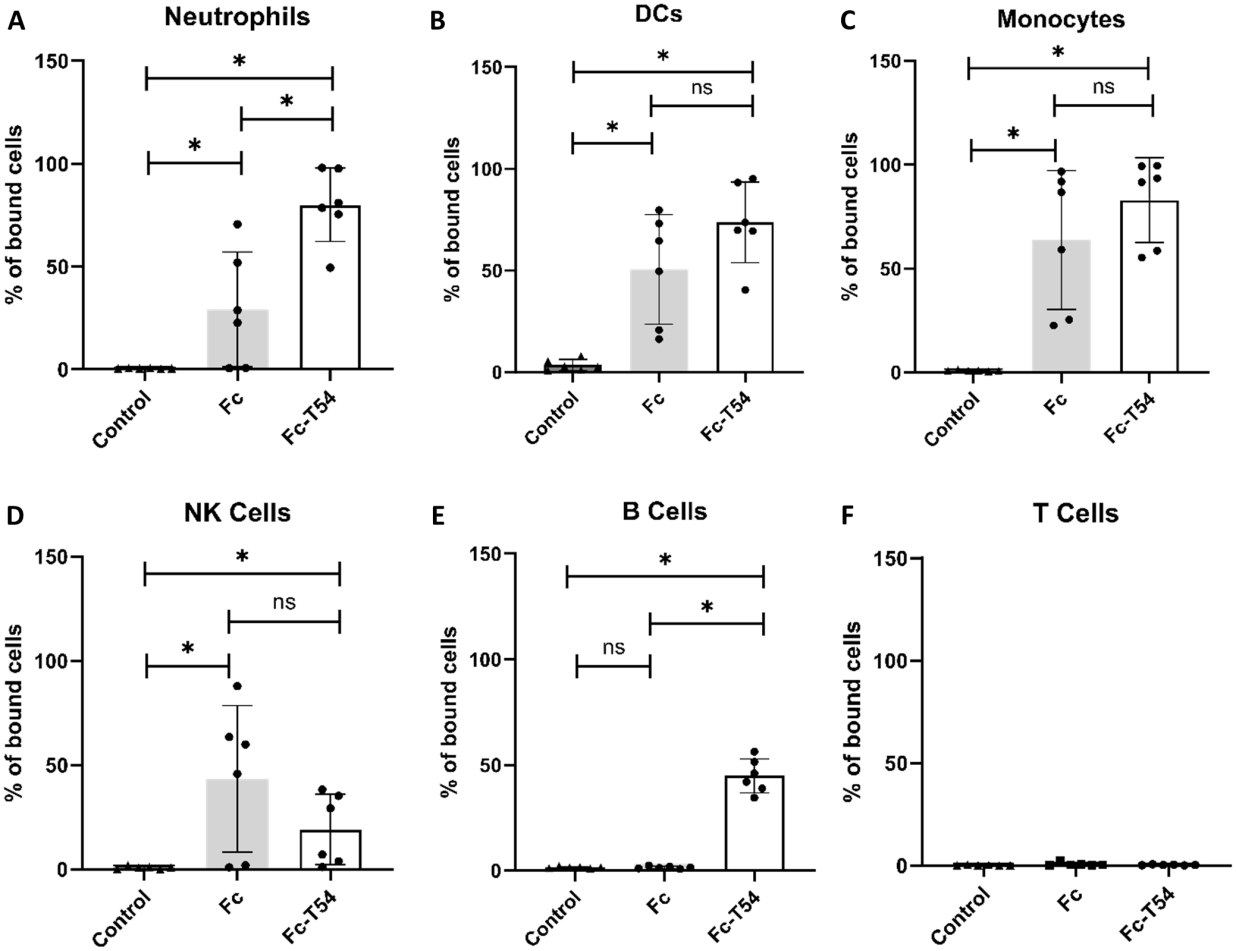
Binding of Fc-T54 and Fc to immune cells within leukocytes from whole blood. Leukocytes from human whole blood were incubated in the presence or absence of biotinylated Fc-T54 or Fc for 30 minutes at 4°C. After incubation, cells were washed and stained with a panel of lineage-specific antibodies to identify neutrophil **(A)**, dendritic cells (DCs) **(B)**, monocytes **(C)**, natural killer cells (NK cells) **(D)**, B lymphocytes (B cells) **(E)**, and T lymphocytes (T cells) **(F)**. Fc-T54 and Fc binding to these immune cell subsets was detected using fluorescent streptavidin. The percentage of fluorescent streptavidin-positive cells was plotted for each parent population. Data are presented as the mean ± SD of 3 independent experiences including a total of 5 donors, paired t test, *p*< 0,05 (*), ns: non-significant.

To assess whether T54 coupling also affects the ability of Fc to bind to human FcγR-expressing immune cells, Fc-T54 and Fc were compared for their binding to human leukocytes from whole blood (**Figure 3**). Fc and Fc-T54 did not interact with T lymphocytes that did not express FcγRs (**Figure 3E**). Fc also did not bind to B lymphocytes. In contrast, about 41% of the B cells were bound by Fc-T54, suggesting that T54 coupling increases binding to FcγRIIb on these cells. Fc was capable of binding NK cells, neutrophils, monocytes, and DCs in agreement with the expression of FcγRs with higher affinity than FcγRIIb on the different cell types (**Figures 3A–D**) (12). Compared with Fc, the proportion of neutrophils interacting with Fc-T54 was higher, suggesting that T54 coupling increases binding to FcγRs in these cells. The difference between Fc and Fc-T54 was not significant for DCs and monocytes. For DCs, we assumed that this might be related to their low amount (<1%) in leukocytes as compared to neutrophils (>50%), which would be mostly bound by the fusion protein. Interestingly, we found a significant difference between Fc and Fc-T54 for DCs and monocytes when peripheral blood mononuclear cells were used (**Supplementary Figure 1**). Surprisingly, while we observed higher binding of the fusion protein to soluble FcγRIIIa by ELISA (**Figure 2C**), we did not find such a difference with NK cells that express FcγRIIIa. The reason for this behavior remains elusive.

### The Fc-T54 fusion protein modulates the activity of leukocytes from whole blood

**Figure 4:**
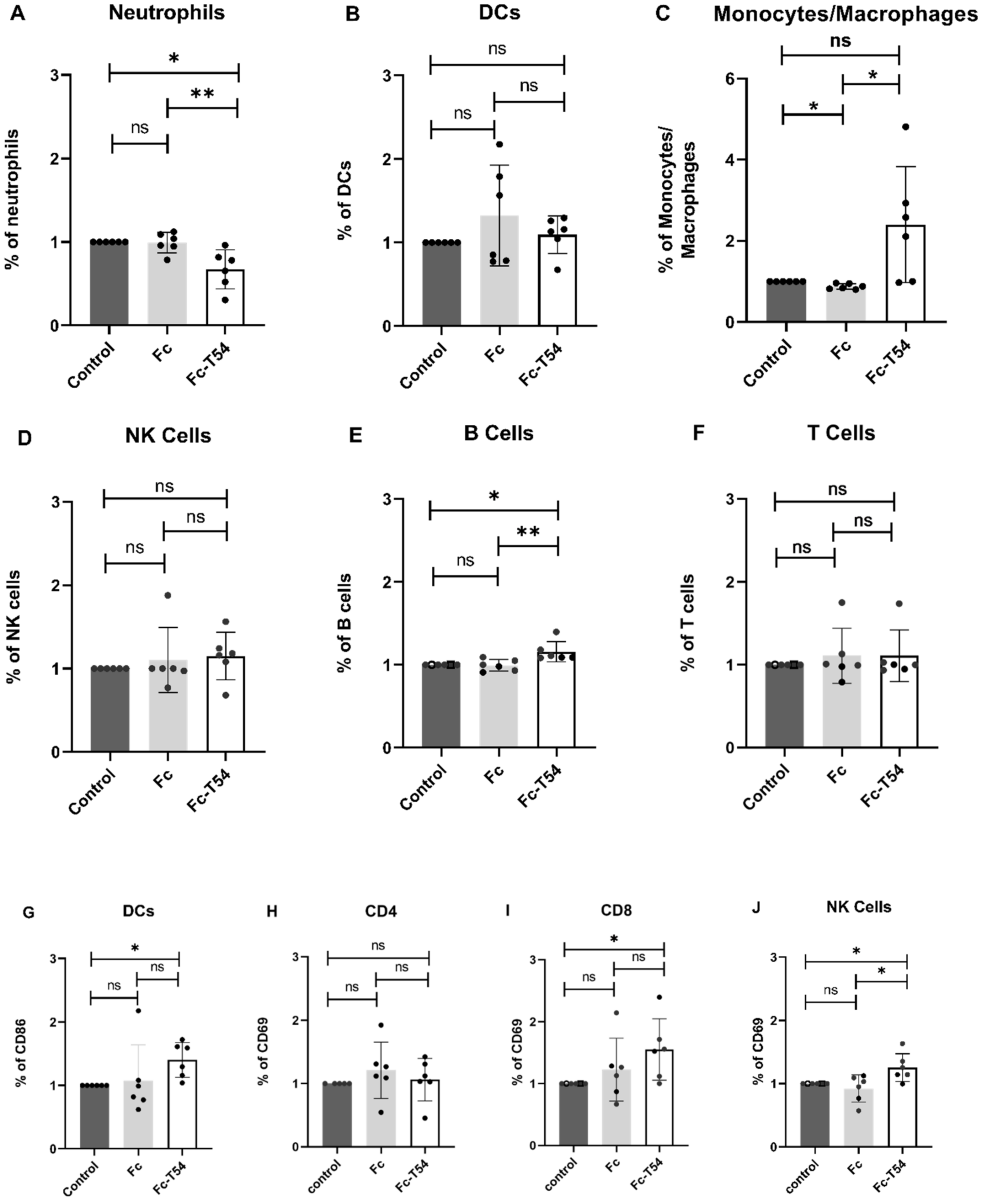
Fc-T54 impact on human immune cell subpopulations. Leukocytes from human whole blood were incubated for 3 days in the presence or absence of Fc-T54 or Fc. Cells were washed and stained with a panel of lineage-specific antibodies to identify neutrophil **(A)**, DCs **(B)**, monocytes/macrophages **(C)**, NK cells **(D)**, B cells **(E)**, and T cells **(F)**. The activation status was evaluated by quantifying CD69 expression for CD4+ T cells **(H)**, CD8+ T cells **(I)** and NK-cells **(J)**. CD86 expression was used for DCs **(G)**. The percentage of cells and of activated cells was calculated relative to the total population of each cell type. Data are shown as mean ± SD of 3 independent experiences, including a total of 6 donors, paired t test, *p* < 0,05 (*), *p* < 0,01 (**), ns: non-significant.

To assess whether the fusion protein affected immune cells, we incubated *in vitro* leukocytes from human whole blood for three days in the presence or absence of either Fc-T54 or Fc. We then determined the proportion of different subpopulations using flow cytometry. As shown in **Figure 4**, Fc-T54 reduced the proportion of neutrophils, whereas Fc had no such effect (**Figure 4A**). Compared to Fc, the fusion protein increased the percentage of B lymphocytes and monocytes/macrophages but had no effect on DCs, NK cells, and T-cells (**Figures 4B–F**). To assess whether Fc-T54 could nevertheless impact the activity of the three latter populations, we assessed the expression of i) the CD69 early activation marker on NK cells, CD4+, and CD8+ T-cells, and ii) the CD86 costimulatory molecule on DCs. We did not find increased expression of CD69 in CD4+ T cells (**Figure 4H**). In contrast, compared to the control, we found upregulated CD69 expression on CD8+ T-cells (**Figure 4I**) and NK cells (**Figure 4J**) as well as a higher CD86 expression on DCs (**Figure 4G**), indicating that the fusion protein enhances activation of these immune cells.

### The Fc-T54 fusion protein interacts more efficiently than Fc with mouse dendritic-cells via a mechanism involving FcγR-mediated binding and HS-binding ability

**Figure 5:**
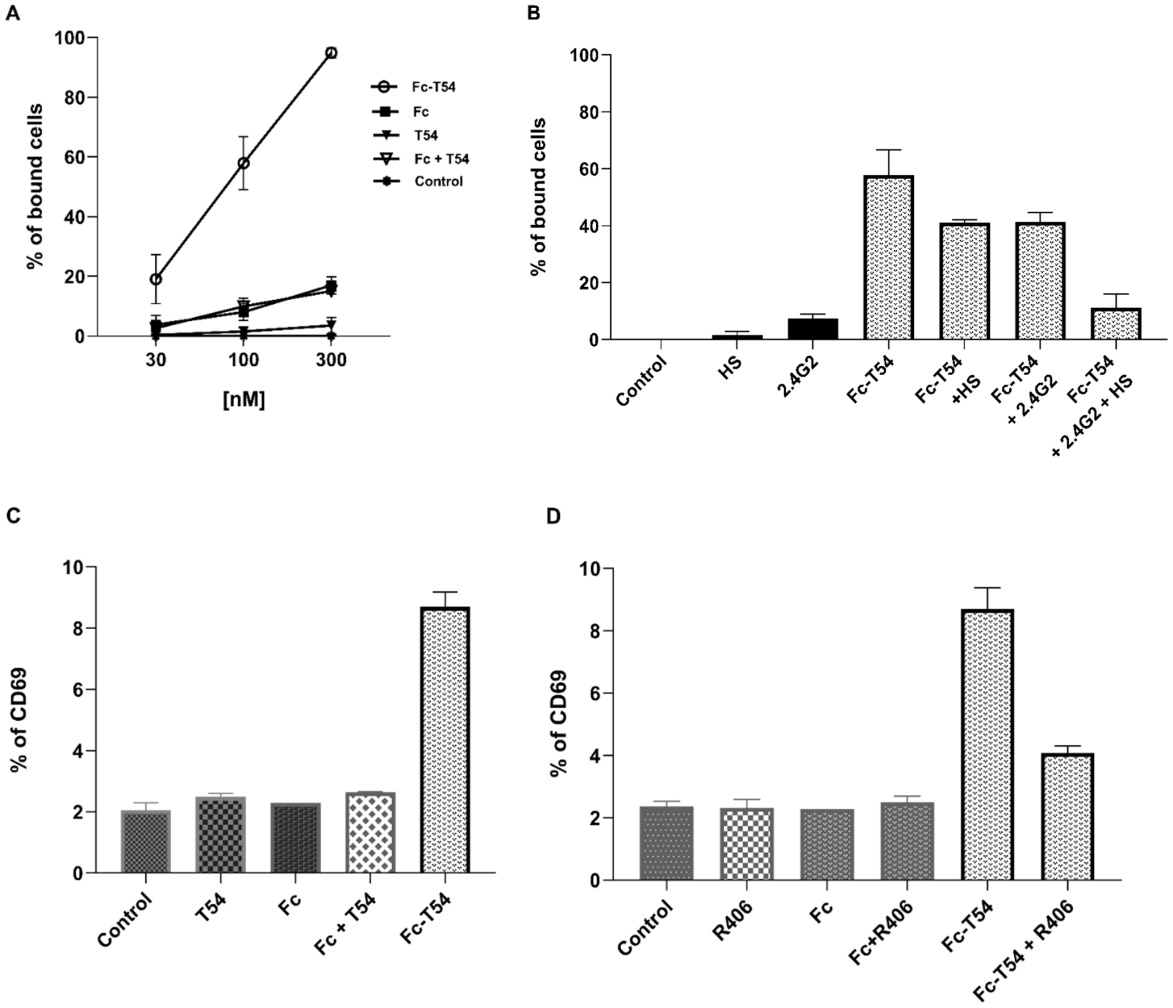
Fc-T54 ability to bind and activate mouse DCs. **(A)** Fc-T54 and Fc binding to DCs. JAWSII DCs were incubated with Fc-T54, Fc, T54 or Fc + T54 for 30 min in 4 °C. Then, cells were labelled with a fluorescent anti-human IgG Ab, and binding was assessed by flow cytometry. The percentage of fluorescent cells was calculated relative to the total number of live cells. **(B)** Inhibition of Fc-T54 binding to DCs. Fc-T54 was incubated alone or in the presence of a Fc receptor-blocking Ab (2.4G2), soluble heparan sulfate (HS), or a combination of 2.4G2 and HS. Binding was analyzed as in **(A, C).** To assess Fc-T54-mediated activation of DCs, JAWSII cells were incubated with or without Fc-T54, Fc, Fc + T54, and T54. After 24 hours, cells were labelled with a fluorescent anti-CD69 Ab. The percentage of CD69-positive cells was plotted relative to the number of live cells. **(D)** DCs were pretreated with the syk pathway inhibitor R406. Thirty minutes later, cells were incubated with or without Fc-T54 and Fc. After 24 hours, cell activation was assessed as in C.

As human IgG1 can recognize mouse FcγRs (30), to validate the use of Fc-T54 in murine models, we assessed its ability as well as that of Fc to bind mouse FcγR-expressing immune cells. For this, we used a mouse DC-line named JAWSII. We found that Fc or a mixture of Fc and T54 peptide bound to less than 20% of DCs, while Fc-T54 interacted with around 90% of cells (**Figure 5A**). Next, to assess whether this enhanced binding depends on the FcγR-binding and HS-binding ability of T54, we evaluated the impact of an anti-FcγRII/III ab (2.4G2 Ab) and soluble HS on Fc-T54 binding to JAWSII cells. We found an about 30% lower interaction in the presence of 2.4G2 Ab or HS (**Figure 5B**), indicating that FcγR and HS binding contribute to Fc-T54 binding. In addition, we observed about 80% inhibition when anti-FcγRII/III ab and HS were incubated together, indicating a potentiated impact on binding. Altogether, these results demonstrate that the Fc-T54 fusion protein interacts more efficiently than Fc with mouse DCs and that this increased interaction proceeds via a mechanism involving low-affinity FcγRs and HS-binding ability.

### The Fc-T54 fusion protein activates i) mouse dendritic cells *in vitro* more efficiently than Fc, through a mechanism that involves the Syk tyrosine kinase signaling pathway, ii) DC1 and CD4+ T cell in mice

To assess whether T54 covalent coupling to Fc increases the ability to trigger mouse DC activation, we assessed CD69 expression in the JAWSII DC-line after 24 h incubation with Fc-T54, T54, Fc, and Fc + T54. We found a tiny impact, if any, on this early activation marker with molecules T54, Fc, and Fc + T54 (**Figure 5C**). In contrast, we observed a four-fold higher expression in the presence of Fc-T54. These results indicate that T54 covalent coupling increases the ability of Fc to trigger mouse DC activation. Next, as FcγR-mediated activation proceeds through the Syk-tyrosine kinase pathway (31), we investigated its contribution using a Syk-pathway inhibitor, named R406. We incubated the fusion protein with or without R406 and assessed CD69 expression. We found an approximately 70% decrease in Fc-T54-mediated CD69 expression in the presence of R406, indicating the contribution of the Syk signaling pathway to activation (**Figure 5D**). Altogether, these results demonstrate that T54 covalent coupling to Fc indeed increases the ability to trigger mouse DC activation and that this increased activation proceeds via a mechanism involving the Syk signaling pathway.

We next administered a low dose of Fc-T54 (20 pmol per injection) via the subcutaneous route to C57BL/6 mice to assess whether the compound could induce immune cell activation *in vivo*, particularly DCs. Compared with the PBS-injected control group, we did not observe an increase in the proportion of activated DC2 cells in the spleen or peripheral blood of Fc-T54–treated animals (**Supplementary Figures 2F, N**). In contrast, a higher proportion of activated DC1 cells was detected in the spleen (**Supplementary Figure 2E**). Additionally, we observed an increased proportion of activated CD4+ T cells in the blood of Fc-T54–treated mice compared with controls (**Supplementary Figure 2O**). Together, these findings indicate that the compound is capable of inducing immune activation in healthy animals.

### Subcutaneous administration of low doses of either a mouse Fc-T54 surrogate or Fc-T54 effectively controls the growth of tumors exhibiting diverse immunosuppressive profiles

In an initial series of experiments, to avoid biases associated with the potential induction of anti-drug antibodies by Fc-T54 or differences in binding to mouse FcγRs (30), we assessed tumor control efficacy using a surrogate for Fc-T54, termed mFc-T54, in which the human Fc domain was replaced with a murine IgG2a Fc. To validate the use of this surrogate, we performed preliminary experiments demonstrating that mFc-T54 interacts with and activates JAWSII dendritic cells more efficiently than mFc alone (**Supplementary Figure 3**). We then evaluated the effect of low-dose subcutaneous injections of mFc-T54 (20pmol/injection) in four syngeneic tumor models.

**Figure 6:**
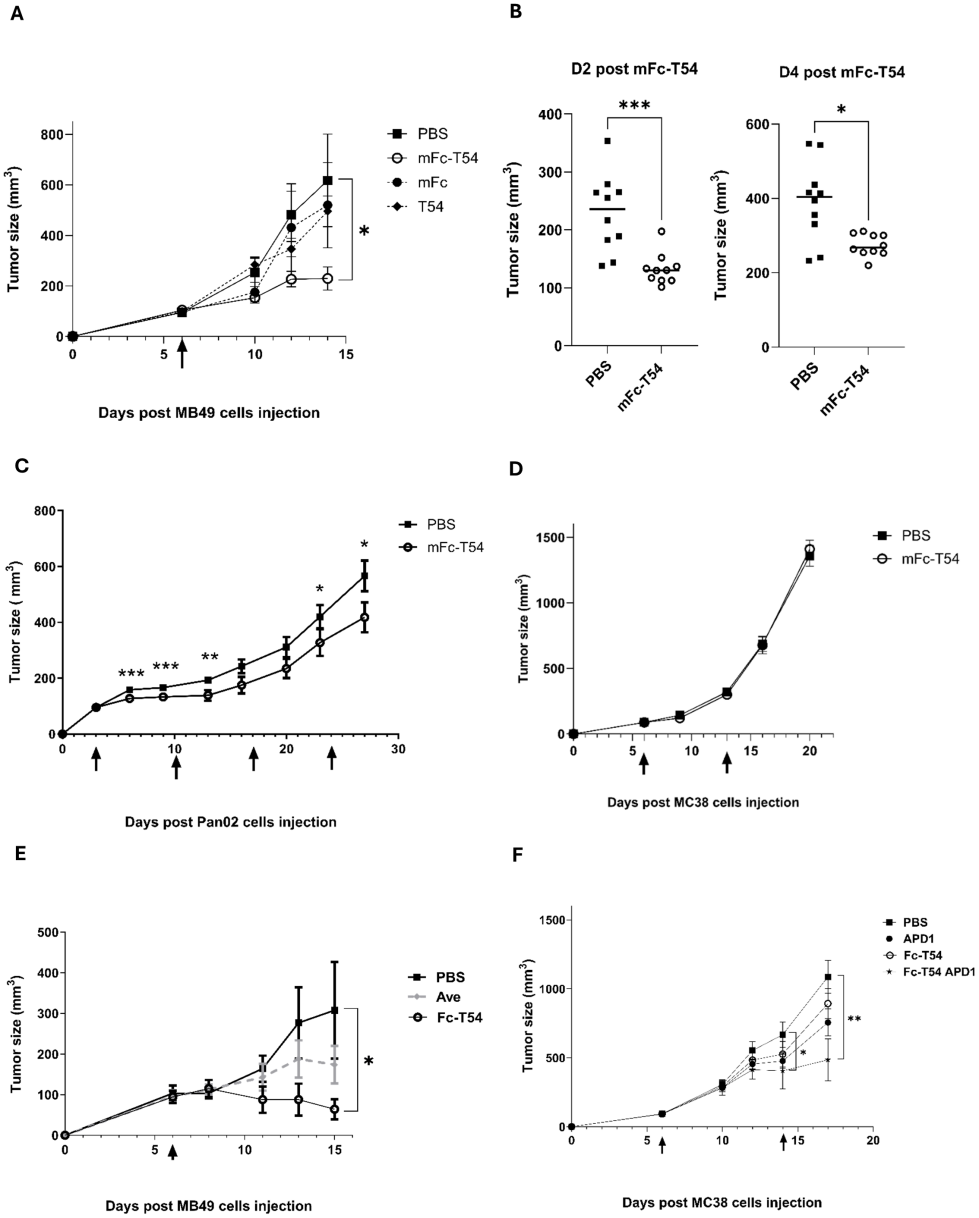
mFc-T54 and Fc-T54 reduce tumor growth in different syngeneic tumor models. **(A)** Four groups of C57BL/6 mice (n=7 to 9 per group) were injected subcutaneously (s.c.) with 1× 10^6^ MB49 cells. Six days later, the groups were injected s.c. with mFc-T54, mFc, T54 and PBS, respectively. **(B)** Two groups of C57BL/6 mice (n=10 per group) were injected s.c. s.c. with 0.2 × 10^6^ B16F10 cells. Ten days later, one group received mFc-T54, another received PBS. Tumor volume at days two and four is presented. **(C)** Two groups of C57BL/6 mice (n=10 per group) were injected s.c. with 3 × 10^6^ Pan02 cells. Three, ten, seventeen and twenty-four days later, one group received mFc-T54, another received PBS. Tumor volume at days two and four is presented. **(D)** Two groups of C57BL/6 mice (n=10 per group) were injected subcutaneously with 1 × 10^6^ MC38 cells. Six and thirteen days later, one group received mFc-T54, another received PBS. **(E)** Three groups of C57BL/6 mice (n=5 per group) were injected s.c. with 0.5 × 10^6^ MB49 cells. Six days later, one group received Fc-T54, another received the Avelumab anti-PD-L1 biosimilar Ab, and the third received PBS. **(F)** Four groups of C57BL/6 mice (n=5 to 8 per group) were injected subcutaneously with 0.5 × 10^6^ MC38 cells. Six and fourteen days later, one group received Fc-T54 s.c., another received the anti-PD-1 Ab i.p., the third Fc-T54 and anti-PD-1 Ab, the fourth received PBS. **(A–F)** The arrow(s) indicate the day(s) of protein injection. Tumor growths were monitored every two days using a caliper. Mice were euthanized at the end of the experiment or when they reached the protocol-ethical defined limits. Data are presented as the mean ± SEM. Mann-Whitney test, *p* < 0,05 (*), p < 0,01 (**), p < 0,001 (***).

The first model used was MB49 bladder cancer, which displays ‘desert’ characteristics, featuring a low proportion of T lymphocytes and a high proportion of myeloid-derived suppressor cells (MDSCs) (32), yet can still be controlled by standard anti-immune checkpoint antibodies (33). In this model, mFc-T54 inhibited tumor growth, whereas mFc and T54 alone were ineffective, indicating that only the fusion protein had an antitumor effect (**Figure 6A**). Next, we employed a second ‘desert’ model: the highly aggressive B16F10 melanoma, characterized by limited immune cell infiltration (34). We observed a significant impact on tumor growth on D2 and D4 after mFc-T54 injection (**Figure 6B**). Here, mFc-T54 treatment significantly inhibited tumor growth on days 2 and 4 post-injection (**Figure 6B**). However, this effect did not persist over time (data not shown), suggesting that the treatment was primarily effective during the early tumorigenic phase in this highly immunosuppressed model. We also tested the Pan02 pancreatic tumor model, which displays ‘excluded’ features with immune infiltration at the tumor periphery, high abundance of immunosuppressive cells (Tregs, MDSCs, and TAMs), and low CD8+ T cell content (35). In this model, mFc-T54 treatment significantly reduced tumor growth (**Figure 6C**). Finally, we evaluated the MC38 colorectal cancer model, which exhibits inflammatory characteristics with high immune cell infiltration and a low proportion of immunosuppressive cells (34, 36). In this model, mFc-T54 treatment had no discernible effect on tumor growth (**Figure 6D**).

Next, we used the MB49 model to evaluate the effect of a low-dose (20 pmol) subcutaneous injection of human Fc-T54 in comparison to an anti-immune checkpoint antibody administered at a standard dose for mice (100 µg, 667 pmol). To enable this comparison, we leveraged the ability of human anti-PD-L1 antibody drugs to cross-react with the mouse PD-L1 (37). We selected avelumab, commonly used in bladder cancer patients, and employed an avelumab (Ave) biosimilar for the study. As shown in **Figure 6E**, Fc-T54 treatment led to a reduction in tumor size, and this effect was greater than that achieved with a 33-fold higher dose of Ave.

Finally, we assessed the effects of Fc-T54 on the MC38 model. Given the sensitivity of MC38 cells to immune checkpoint blockade (38) we also tested whether our immunotherapy could synergize with anti-PD-1 antibody treatment. In this inflamed tumor context, neither Fc-T54 nor anti-PD-1 antibody monotherapy significantly affected tumor growth (**Figure 6F**). However, combination treatment resulted in a marked reduction in tumor size, indicating a synergistic therapeutic benefit.

To obtain a preliminary assessment of the safety profile of Fc-T54, two *in vivo* studies were performed. First, the murine surrogate mFc-T54 was used to evaluate its potential to induce anti-drug antibody (ADA) responses in healthy mice. Repeated subcutaneous administration of mFc-T54 (20 pmol per injection, three injections at one-week intervals) did not lead to detectable ADA formation, as Fc-T54–specific IgG titers remained unchanged in sera collected up to 35 days after the first injection (**Supplementary Figure 4**). Second, the ability of Fc-T54 to induce cytokine release was assessed *in vivo*. A single administration of Fc-T54 did not trigger significant cytokine production, as multiplex assay analysis of serum collected at one and seven days post-injection revealed no significant differences in cytokine levels compared with PBS-treated controls (**Supplementary Figure 5**). Collectively, these results suggest a favorable safety profile, with no evidence of overt immunogenicity or cytokine induction in healthy mice.

### Fc-T54 injection changes the pattern of immune cells infiltrating tumor microenvironment

**Figure 7:**
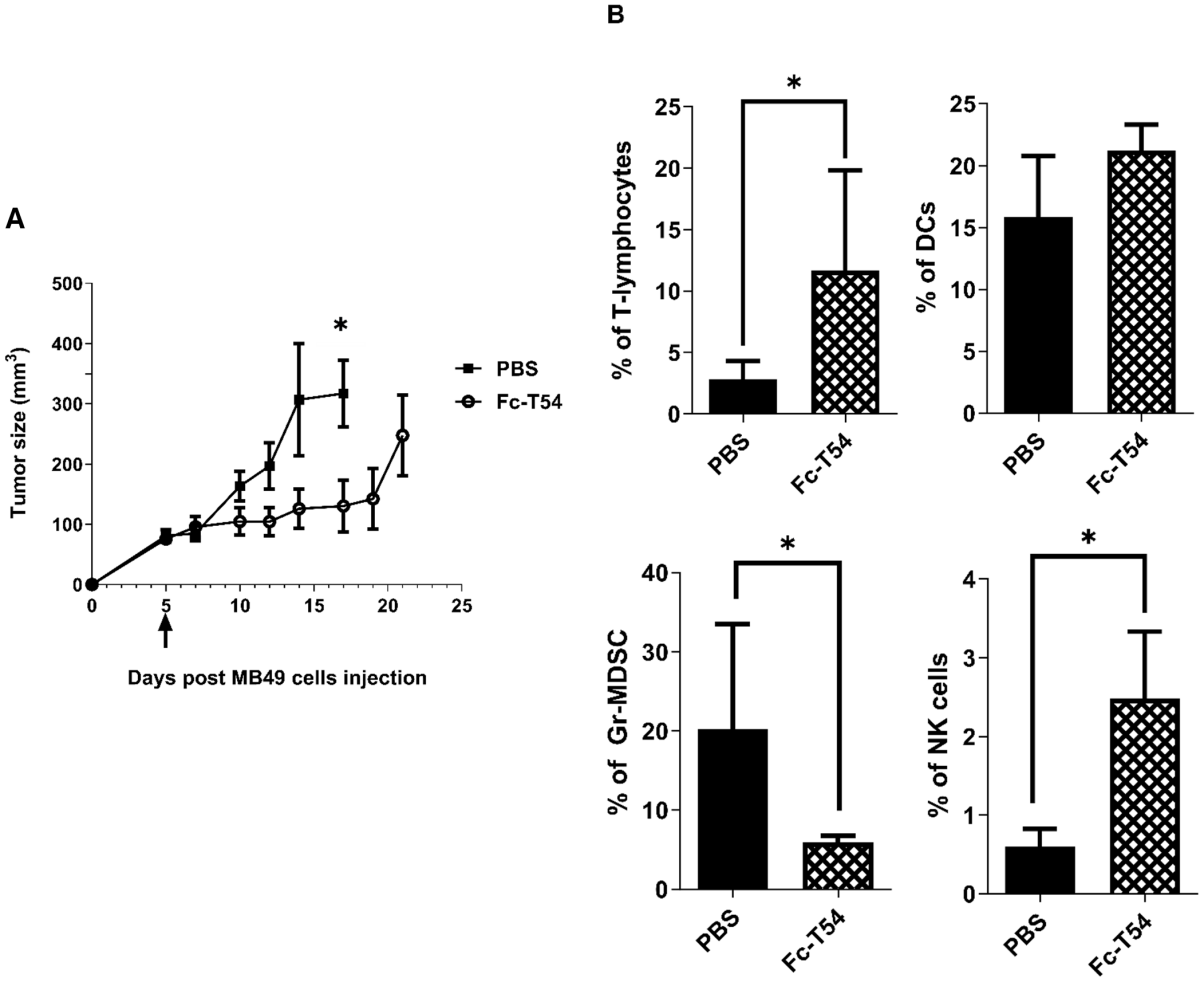
Fc-T54 injection changes the pattern of immune cells infiltrating MB49 tumor microenvironment. **(A)** Mice injected with MB49 cells exhibit slower tumor growth when subsequently treated with Fc-T54. Two groups of C57BL/6 mice (n=5 and 6 per group) were injected s.c. with 1 M MB49 cells. Five days later, one group was injected with Fc-T54, another with PBS as control. Tumor growth was monitored using a caliper. **(B)** Distinct immune cells infiltration in MB49 injected mice treated with Fc-T54 or PBS. Tumors were collected from mice injected with PBS or Fc-T54, and cells were labelled with fluorescent specific antibodies for T-lymphocytes, DCs, Gr-MDSC-cells and NK-cells, respectively. The proportion of each population is represented among total live cells. Data are presented as the mean ± SEM. Mann-Whitney test, *p < 0.05.

To assess whether the effect of Fc-T54 on tumor growth is related to a change in the profile of immune cells infiltrating the TME, we selected the MB49 syngeneic model, which has a TME with a significant proportion of MDSCs, DCs, T cells, and NK cells (32). Given that Fc-T54 injection into mice implanted with the amount of MB49 cells commonly used by research groups (i.e., 0.5 M) caused a reduction in tumor size (see **Figure 6D**) that did not allow the collection of enough cells for accurate immunophenotyping, we injected a two-fold higher amount of cells to obtain a lower tumor control. These conditions fit well, since mice injected with Fc-T54 control tumor growth without a size decrease (**Figure 7A**). Therefore, we collected tumors from the control and treatment groups for immunophenotyping. As shown in **Figure 7B**, as compared to the control group, the TME of animals injected with Fc-T54 exhibited four times fewer MDSCs, an insignificant increase in DC, and an approximately three-fold increase in T- and NK-cells. Therefore, these data indicate that the fusion protein makes the TME more prone to tumor control.

## Discussion

We engineered our Fc-T54 fusion protein to modulate myeloid cell activity through the co-engagement of FcγRs and heparan sulfate proteoglycans (HSPGs) (**Supplementary Figure 6**). Compared to Fc, Fc-T54 exhibited reduced binding to FcγRI and enhanced interactions with low-affinity FcγRIIa, FcγRIIb, and FcγRIIIa. Previous structural studies indicated that complex formation between the Fc moiety of IgG1 and the different FcγRs is closely related to significant contributions from the lower hinge region and the CH2 domains. However, the nature and strength of these interactions vary due to distinct structural features, such as the presence of a shorter FG loop in the D2 domain of FcγRI and the influence of glycosylation on the immunoglobulin CH2 domain, FcγRIIs, and FcγRIIIa (39). Since Fc-T54 is a weaker binder than Fc for high-affinity FcγRI and a stronger binder for low-affinity FcγRs, we hypothesized that C-terminal fusion of T54 to Fc may induce long-range structural effects in the CH2 domains, thereby differentially modulating affinity depending on the FcγR subtype. The coupling of T54 enables Fc-T54 to bind heparin, a representative molecule of the HS family. Thus, the fusion protein was capable of simultaneously interacting with both FcγRs and heparin. This dual-binding capability suggests that Fc-T54 could serve as a relevant molecule for simultaneously engaging FcγRs and HSPG coreceptors on immune cells expressing FcγRs, particularly myeloid cells.

Experiments performed with leukocytes from human whole blood showed that Fc-T54 has a cell-binding pattern similar to that of Fc, strongly suggesting that its interaction is primarily mediated by binding to FcγRs. Compared to Fc, the fusion protein interacts more strongly with the predominant myeloid population, neutrophils, indicating that coupling with the T54 domain enhances binding. Fc-T54 did not exhibit significantly increased binding to monocytes, NK cells, NKT cells, or DCs. For the latter population, this may be due to its low proportion relative to neutrophils, as we found a significantly higher number of DCs bound by Fc-T54 compared to Fc when using human peripheral blood mononuclear cells (PBMCs) that were depleted of neutrophils (**Supplementary Figure 1**). Furthermore, we observed that the fusion protein binds more effectively than Fc to a murine DC-line, and this enhancement depends on the FcγRII/III and HS-binding capacity. Collectively, these data indicate that Fc-T54 has an enhanced ability to bind to FcγR-expressing myeloid cells.

*In vitro*, Fc-T54 affected the immune cells to which it binds more strongly. Incubation with Fc-T54 reduced the proportion of neutrophils, whereas incubation with Fc had no effect, suggesting the induction of inhibitory signals. The underlying mechanisms remain to be clarified, as these cells express FcγRIIa, FcγRIIc, and FcγRIIIb, and to a lesser extent, inhibitory FcγRIIb (14). Fc-T54 increased the proportion of B lymphocytes and macrophages, indicating that it may induce activating signals. Blood dendritic cells (DCs) show upregulated CD86 expression, reflecting enhanced activation. Increased activation was observed in a murine DC line. This enhancement was abrogated by a Syk tyrosine kinase pathway inhibitor, confirming the involvement of the FcγR signaling cascade (31). Collectively, these findings support the hypothesis that activation is modulated by the co-engagement of FcγRs and an HSPG coreceptor, thereby amplifying FcγR-mediated cell signaling.

The enhanced modulatory activity resulting from Fc**γ**R/HSPG coreceptor engagement prompted us test low-dose Fc-T54 (20 pmoles, 30–60 times than of typical ICIs doses (40)) in rodents. Given its ability to activate DCs, we prioritized the subcutaneous route due to the high density of DCs in the skin (41, 42). We first assess the impact of this regimen in healthy mice. We observed an increased proportion of activated DC1 in the spleen and activated CD4 T cells in the blood following subcutaneous Fc-T54 administration, whereas DC2 activation remained unchanged. This pattern is consistent with a preferential engagement of cross-presenting DC1, which are key orchestrators of T cell–mediated antitumor immunity, and suggests that Fc-T54 can initiate a productive immune response. Then we assessed Fc-T54 impact in multiple murine syngeneic tumor models. This approach proved effective as Fc-T54 induced tumor control in three models. These findings support the low-volume subcutaneous injection of Fc-T54 in clinical trials, an approach generally avoided for ICIs due to high-dose requirements. However, recent studies have shown that subcutaneous ICI administration can match intravenous efficacy, while enhancing patient comfort and quality of life (43, 44).

Certain ICIs can reshape the TME by increasing T cell infiltration and reducing immunosuppressive cells, such as Tregs and MDSCs, thereby converting it into a more immunologically active milieu (45). Targeting MDSCs or reprogramming TAMs toward a pro-inflammatory phenotype enhances antitumor immunity (46, 47). The activation and recruitment of DCs are also key factors in initiating and sustaining anti-tumor immune responses (48). Collectively, these studies illustrate that modulating the immune cell composition of the TME is a validated therapeutic approach for controlling tumor growth (49). In line with these findings, our Fc-T54 immunotherapy was capable of remodeling the TME of the MB49 syngeneic model (32) by decreasing the proportion of MDSCs and increasing its T-lymphocyte and NK-cell content. Thus, Fc-T54 transforms an immunosuppressive niche into one more amenable to tumor control.

Fc-T54 displays broad and versatile antitumor efficacy across syngeneic mouse models representing immune-desert, immune-excluded, and immune-inflamed tumor phenotypes. In these desert models, Fc-T54 overcomes pronounced local immunosuppression, notably by controlling tumor growth in MB49, where anti-PD-L1 is effective, and delaying early progression in the highly aggressive, rapidly growing B16F10 model, which is resistant to checkpoint inhibition. In the immune-excluded Pan02 model, Fc-T54 reduced tumor burden, suggesting its capacity to bypass exclusion barriers. In contrast, Fc-T54 alone had no effect on the immune-inflamed MC38 model but combined synergistically with anti-PD-1 therapy. Altogether, these findings highlight Fc-T54 as a flexible immunotherapeutic option for tumors with diverse immunosuppression, growth dynamics, and ICI responsiveness, potentially expanding treatment avenues for ICI-resistant, desert/excluded tumors and in combination regimens for inflamed tumors.

Translating Fc-T54 toward clinical application will require careful consideration of several key aspects, including immunogenicity, pharmacokinetics, toxicity and the scalability of manufacturing. In particular, the presence of an HSPG-binding moiety fused to the Fc region underscores the need to comprehensively characterize Fc-T54 exposure, tissue distribution, and persistence following subcutaneous administration, as well as to ensure that the production process is robust and amenable to large-scale implementation. Preliminary studies in mice indicate that the murine Fc-T54 surrogate (mFc-T54) is not immunogenic and that Fc-T54 does not trigger detectable cytokine release, suggesting a low propensity for systemic inflammatory toxicity. These encouraging observations will nonetheless need to be corroborated in dedicated preclinical safety and toxicology studies as well as the scalability of manufacturing, which will be essential to support the progression of Fc-T54 into first-in-human trials.

## Data Availability

The datasets presented in this study can be found in online repositories. The names of the repository/repositories and accession number(s) can be found below: https://doi.org/10.5281/zenodo.17465975, 10.5281/zenodo.17465976.
