# Supplementary material for: A low-dose immunotherapy targeting Fc gamma Receptors and heparan sulfate proteoglycan to impact myeloid cells and control cancers with diverse immunosuppressive profiles
Source: Front Immunol. 2026 Jul 23;17:1734778. doi: 10.3389/fimmu.2026.1734778 (PMC13443304; doi:10.3389/fimmu.2026.1734778)
Supplement: Supplementary file 1 [file DataSheet1.docx]

Figure S1 :

**Figure S1. Binding of Fc-T54 and Fc to immune cells within PBMCs.** PBMCs from human whole blood were incubated in the presence or absence of biotinylated Fc-T54 or Fc for 30 minutes at 4°C. Then, cells were washed and stained with a panel of lineage-specific antibodies to identify dendritic cells (cDC and pDC), monocytes, natural killer cells (NK), natural killer T cells (NKT), B lymphocytes (B cells), and T lymphocytes (T cells). Fc-T54 and Fc binding to these immune cell subsets was detected using fluorescent streptavidin. The percentage of fluorescent streptavidin-positive cells was plotted for each parent population. Data are presented as the mean ± SD of 5 independent experiences, paired t test (*p < 0.05; **p < 0.01; ns, non‑significant).

**Supplementary Table 1.** List of human antibodies and staining reagents for flow cytometry.

| **Fluorophore** | **Antigen** | **Clone** | **Catalog Number** | **Vendor** | **Dilution** |
| --- | --- | --- | --- | --- | --- |
| APC Cy7 | CD3 | UCHT1 | 300426 | Biolegend | 100 |
| BV711 | CD56 | NCAM16.2 | 563169 | BD | 100 |
| CF594 | CD19 | HIB19 | 562294 | BD | 100 |
| FITC | HLA-DR, DP, DQ | Tü39 | 361706 | Biolegend | 1000 |
| APC | CD11B | M1/70 | 101212 | Biolegend | 100 |
| AF700 | CD66B | G10F5 | 305114 | Biolegend | 100 |
| BV421 | CD15 | HI98 | 567008 | BD | 100 |
| BV605 | CD14 | M5E2 | 564054 | BD | 100 |
| Pe CY7 | CD11c | 3.9 | 301608 | Biolegend | 200 |
| PECY7 | CD56 | B159 | 557747 | BD | 100 |
| APC | CD20 | 2H7 | 560853 | BD | 100 |
| PerCP-Cy5.5 | CD123 | 6H6 | 306016 | Biolegend | 100 |
| APC | CD303 | 201A | 354206 | Biolegend | 100 |
| FITC | CD8 | RPA-T8 | 301060 | Biolegend | 200 |
| BV421 | CD69 | FN50 | 310930 | Biolegend | 100 |
| Pe CY7 | CD4 | SK3 | 557852 | BD | 100 |
| PerCPCy5-5 | CD16 | B73.1 | 360712 | Biolegend | 100 |
| CF594 | CD86 | 2331 (FUN-1) | 562390 | BD | 100 |
| PE | CD66b | G10F5 | 561650 | BD | 100 |

**Supplementary Table 2.** List of mouse antibodies and staining reagents for flow cytometry.

| **Fluorophore** | **Antigen** | **Clone** | **Catalog Number** | **Vendor** | **Dilution** |
| --- | --- | --- | --- | --- | --- |
| APC | CD11b | M1/70 | 101212 | Biolegend | 800 |
| PE | Ly6C | HK1.4 | 128007 | Biolegend | 100 |
| BV785 | Ly6G | 1A8 | 127645 | Biolegend | 200 |
| PECy7 | CD11C | N418 | 117318 | Biolegend | 200 |
| FITC | I-A/I-E | 2G9 | 553623 | BD | 1000 |
| PeCy7 | NK1-1 | PK136 | 108714 | Biolegend | 200 |
| APC Cy7 | CD3 | 17A2 | 100222 | Biolegend | 200 |
| PE-Dazzle 594 | CD45 | 30-F11 | 103146 | Biolegend | 800 |

**A**

**B**

**C**

**D**

**E**

**F**

**G**

**H**

**I**

**J**

**K**

**L**

**M**

**N**

**O**

**P**

**Figure S2: Fc-T54 activates DC1 and CD4+ T cell in healthy mice:** Two groups of C57BL/6 mice (n = 6 per group) were injected subcutaneously with 20 pmoles of Fc‑T54. Five days later, spleen and whole blood samples were collected for immune phenotyping by flow cytometry. The first eight panels (A–H) correspond to analyses performed in the spleen, and the following eight (I–P) in peripheral blood. The proportions of DC1, DC2, CD4⁺, and CD8⁺ populations are shown among total live CD45⁺ cells. Activation status was assessed by quantifying CD69 expression on CD4⁺ and CD8⁺ T cells, and CD86 expression on DCs, calculated relative to each respective cell population. Data are presented as mean ± SEM. Statistical significance was determined using the Mann–Whitney test (*p < 0.05; **p < 0.01; ns, non‑significant).

**A**

**B**

**C**

**D**

**E**

**Figure S3. mFc-T54 ability to bind and activate mouse DCs. A:** mFc-T54, Fc-T54, mFc and Fc binding to DCs. JAWSII DCs were incubated with biotinylated mFc-T54, Fc-T54, mFc and Fc for 30min in 4°C. Then, cells were labeled with a fluorescent Streptavidin, and binding was assessed by flow cytometry. The percentage of fluorescent cells was calculated relative to the total number of live cells. **B-E:** mFc-T54-mediated activation of DCs, JAWSII cells were incubated with or without mFc-T54, Fc-T54, mFc and Fc. After 24 hours, supernatants were collected to assess IL-6 secretion (C & E) and cells were labeled with a fluorescent anti-CD69 Ab (B & D). The percentage of CD69 positive cells was plotted relative to the number of live cells.

**Figure S4. mFc‑T54, the murine surrogate of Fc‑T54, is non-immunogenic in healthy mice.** Six C57BL/6 mice were subcutaneously injected with 20 pmoles of mFc‑T54 three times at one‑week intervals. Blood samples were collected 7, 14, 21, and 35 days after the first injection. Serial dilutions of sera were then incubated on plates previously coated with Fc‑T54 (1 µg/ml in PBS) and blocked with BSA (0.3% in PBS). After overnight incubation at 4 °C, plates were washed with PBS‑Tween buffer (0.05%), and a horseradish peroxidase–conjugated polyclonal goat anti‑mouse IgG antibody was added. Thirty minutes later, the plates were washed, and a chromogenic substrate (ABTS) was added. After another 30 minutes, absorbance was measured at 414 nm in each well.

**Figure S5. Fc‑T54 does not trigger cytokine secretion in mice.** Four groups of six C57BL/6 mice were subcutaneously injected with either 20 pmoles of Fc-T54 or PBS. Blood samples were collected at days 1 and 5 post-injection, and cytokine levels were measured using a multiplex cytokine assay. Among the 36 cytokines analyzed, only five were detected within the assay’s quantifiable range: IL-22 (A), Eotaxin (B), IP-10 (C), MCP-3 (D), and RANTES (E).

**Figur****e 6. Schematic overview of Fc-T54 design and proposed mechanism of action.**
**A:** Proposed structural model of Fc-T54 in complex with the extracellular domain of FcγRIIIa (light grey; PDB: 7URU). Fc-T54 was engineered by fusing a FcγR-binding ligand (Fc; blue/green chains) to a six-residue linker (red) and a heparan sulfate-binding ligand (T54; soft brown). **B:** Proposed mechanism of action of Fc-T54 through interactions with heparan sulfate proteoglycans (HSPGs) and Fcγ receptors expressed on myeloid cells, including myeloid-derived suppressor cells (MDSCs) and dendritic cells (DCs), as well as on NK cells.

A

B
